# Body Mass Index in the Early Years in Relation to Motor Coordination at the Age of 5–7 Years

**DOI:** 10.3390/sports5030049

**Published:** 2017-07-07

**Authors:** Arto Laukkanen, Arto J. Pesola, Taija Finni, Arja Sääkslahti

**Affiliations:** 1 Faculty of Sport and Health Sciences, University of Jyväskylä, Jyväskylä 40014, Finland; arja.saakslahti@jyu.fi; 2 Neuromuscular Research Center, Faculty of Sport and Health Sciences, University of Jyväskylä, Jyväskylä 40014, Finland; arto.j.pesola@jyu.fi (A.J.P.); taija.m.juutinen@jyu.fi (T.F.)

**Keywords:** fundamental motor skills, objectively measured physical activity, childhood overweight, early development

## Abstract

Physical activity (PA) and body mass index (BMI) are consistently associated with motor coordination (MC) in children. However, we know very little how BMI in early childhood associates with MC later in childhood. This study investigated associations between BMI in early childhood and BMI, PA, and MC in middle childhood. Children aged 5 to 7 years (*n* = 64, 32 girls) were measured for MC using Körperkoordinationstest für Kinder (KTK) and for moderate-to-vigorous PA (MVPA) using triaxial accelerometers. Prevailing body weight and height were measured, and information on weight and height in early years was based on parental report of child health care report cards. Age-adjusted BMI_z_ scores were calculated on the basis of international growth curve references. Associations and the explained variability of MC were investigated by Pearson correlations and a hierarchical multiple regression analysis. Age and MVPA were found to be significantly associated with MC at middle childhood, in general. BMI_z_ at middle childhood and at ages 4 and 5 years inversely explained 12% (*p* < 0.05), 6% (*p* > 0.05), and 7% (*p* > 0.05) of the variation in MC in girls after adjusting for covariates, respectively. In boys, BMI_z_ scores did not show any trend of association with MC. This study suggests sex-specific mechanisms in the interplay between BMI and motor development in childhood.

## 1. Introduction

Motor coordination (MC) is favorably associated with health markers, such as physical activity (PA), cardiorespiratory fitness, muscular strength, endurance, and healthy weight status throughout childhood [1,2]. While the level of PA has been shown to be a relatively consistent correlate of MC in children and adolescents [3,4], it has been hypothesized that the association becomes stronger over the course of childhood [4,5]. On the other hand, a rapidly growing body of evidence suggests that relative body fatness, typically assessed as height-for-weight ratio by body mass index (BMI, kg/m^2^), plays a significant role in motor development, PA engagement, and fitness throughout childhood [6,7,8]. Essentially, healthy BMI status is hypothesized to feed back to a positive spiral of PA engagement, motor development, and fitness over the course of time [5].

Negative correlations between BMI and MC are reported to vary between 0.05 to 0.49, in 6 to 14-year-olds. The strength of these correlations seem to increase up to the age of 11 years, and then begin to decline [9,10]. However, little is known how BMI development in early childhood years is associated with MC later in childhood. Both BMI and MC are known to track from early to late childhood [11,12,13] and to adulthood [14,15]. In addition, when taking into account that the most excess body weight before puberty is gained before the age of five [16], BMI development during the first five years of life can be hypothesized to be in a dynamic interaction with other health-related markers, such as MC. This is because excessive body mass may hamper participation in PA typical to age and developmental level, and consequently, may inhibit motor development, which, in turn, may feed back to excessive body weight accumulation [5]. In addition, there are likely environmental and behavioral factors mediating the hypothesized relationship between early years’ BMI development and middle childhood’s MC, such as parental education, child’s sex, and habitual PA [17].

Therefore, the aim of the present study was to examine the relationship between BMI in early years and MC at the age of 5–7 years. We hypothesized that BMI in early years predicts MC in middle childhood. We based this hypothesis on the preliminary evidence stating that BMI and MC are strongly associated between the ages of 6 and 14 years [2], and that BMI predicts MC between the ages of 5 and 13 years [6,8].

## 2. Materials and Methods

This study is based on the baseline data of a cluster randomized controlled trial (ISRCTN28668090) [18] examining the effect of a family-based PA intervention on PA and motor development in 4–7-year-old children and on sedentary time in their parents. We received an ethics approval for the study from the Ethics Committee of the Central Finland Health Care District on 25 March 2011 (Dnro 6U/2011), and written informed consent was obtained from all of the parents/guardians (henceforth referred to as the parents) for their child's involvement in the study.

### 2.1. Subjects

Recruitment letters were sent to parents via 601 children attending 21 daycare centers and 454 children attending eight primary schools in a city in central Finland with approximately 133,000 inhabitants. Children attending daycare less than 10 days a month and children with a developmental disorder or other disorders delaying motor development were excluded. At least one parent and a child were required for the family to be included in the study. The recruitment of participants was performed between 1 April 2011 and 30 April 2012. The baseline measurements were conducted for each participant within two weeks, and overall, took place between 2 May 2011 and 2 May 2012.

A total of 101 children were allocated to the study. Altogether, 37 children were excluded from the analyses because of missing early years’ weight (*n* = 25) or height (*n* = 26) data, extremely low birth weight (1270–1300 g, *n* = 2) or birth height (32 cm, *n* = 1), or being under 5 years of age (*n* = 9). The excluded children, except those under 5 years of age, did not significantly differ in terms of MC from the ones involved in the study analyses. In addition, the prevalence of parents’ higher-level education was higher in the present study sample than the mean of the local community (71%/35%).

### 2.2. Motor Coordination

MC of children was assessed by Körperkoordination Test für Kinder (KTK) test battery [19] by one trained researcher (Arto Laukkanen, henceforth referred to as AL). KTK is a product-oriented assessment tool, and it is appropriate for children with a typical developmental pattern as well as for children with brain damage, behavioral problems, or learning difficulties [20]. KTK is a suitable tool for identifying motor problems and impairments in children aged 5–14 years. KTK assesses gross body control and coordination, mainly dynamic balance [13,21], instead of single movement skills. In addition, KTK has been used for the criterion validity studies of other assessment tools, such as Movement Assessment Battery for Children (Movement ABC) [22,23].

From the KTK test battery, the children performed the following four sub-items:

1. Walking backwards (WB) on balance beams (length 3 m, height 5 cm) with different widths of 6.0 cm, 4.5 cm, and 3.0 cm, starting from the widest one. The maximum test score possible was 72 steps, based on three trials per each beam and a maximum of eight successful steps for each trial.
2. Hopping for height (HH), one foot at a time, over an increasing pile of soft mattresses (width 60 cm; depth 20 cm; height 5 cm each). The first, second, or third trial of each height was awarded by three, two, or one point(s), respectively. The maximum test score was 39 points (ground level +12 mattresses) for each leg, resulting in a maximum of 78 points with both legs.
3. Jumping sideways (JS) from side to side over a thin wooden lath (60 cm × 4 cm × 2 cm) on a jumping base (100 cm × 60 cm). Two trials of 15 s were performed and the total of successful jumps was summed.
4. Moving sideways (MS). The children had two identical wooden plates (size 25 cm × 25 cm, height 5.7 cm) and after stepping to one, they had to transfer another one sideways for the next transition. The total of transitions was summed over two 20-s trials. Transitions were performed in the same direction on both trials.

The reliability of the KTK has been shown to be high [19] and robust for maturity in 10-year-old boys [24]. The total score of KTK has been shown to moderately correlate with Movement ABC total score (r = 0.62–0.65) [22] and the Bruininks-Oseretsky Test for Motor Proficiency ( BOT-2) short form total score (r = 0.61–0.64) [23,25]. The KTK protocol has shown moderate to high reliability based on test-retest correlation (r = 0.60–0.99) [10,26,27,28,29], and high reliability based on inter-rater correlation (r = 0.90–0.99). In addition, Cronbach’s alpha between the four items has shown high internal consistency (0.95) [26].

The raw test scores of the KTK test items were transformed into gender- and age-standardized values and into a measure indicating the overall result of the test protocol, according to renewed KTK-reference values [30]. Accordingly, a single MC factor was formed and used in the analysis.

### 2.3. Physical Activity

PA was measured for an average of 5.47 days (11.60 ± 0.91 h/day) in the children using triaxial X6-1a accelerometers with a dynamic range of ±6 g (Gulf Coast Data Concepts Inc., Waveland, MS, USA). Subjects with recordings longer than 480 min on at least three days (two weekdays and one weekend day) were accepted for further analysis [31]. On average, 3.72 (11.72 ± 1.10 h/day) of measured days were weekdays, and 1.75 (11.45 ± 1.06 h/day) were weekend days. The device was carried on the anterior waistline in a firmly worn adjustable elastic belt during waking hours, with the exception of water-based activities and bathing. Verbal and written instructions for accelerometry measurement in children were given individually to parents and teachers at the kindergarten. The time spent at a given intensity category was analyzed using the following cutoff points: sedentary, under 373; light, 373–585; moderate, 585–881; and vigorous, over 881 [32].

Non-wearing time was defined as a 20-min or longer continuous zero signal and was cut out. In addition, midday nap time was cut out from further analysis in children attending kindergarten. Nap times were marked to the diary by the kindergarten teachers.

### 2.4. Weight, Height, and Body Mass Index

Weight (Soehnle Digital personal scale, Soehnle, Germany) and height (wall-attached measuring tape) were measured in the laboratory, school, or kindergarten by a researcher (AL). All children were asked to wear only light clothes and take off their shoes and hats during the measurements.

Weight and height history in the early years of the child was asked from the parents by a questionnaire. In short, parents were asked for their child’s birth weight and height, as well as the child’s weight and height at ages 1, 2, 3, 4, 5, 6 and 7 years. We instructed parents to utilize their child’s health care report card when filling the questionnaire. Nationally, every child takes part in frequent child health care check-ups during the first year of life, and later on, in annual health care check-ups. During these check-ups, qualified public health nurses conduct weight and height measurements, and enter the measurement results into an electric registry and to the report card possessed by the parents. We asked parents to also fill the date of the annual health care check-up in accordance the weight and height information. Parents were instructed not to recall the asked information by heart if the child health care report card was missing or if parts of the information were lacking.

There was considerable variation in the timing of children’s annual health care check-ups. Hence, the children were on average 1.01 ± 0.08 (*n* = 72), 1.96 ± 0.34 (*n* = 66), 2.86 ± 0.29 (*n* = 54), 4.06 ± 0.19 (*n* = 68), 5.06 ± 0.12 (*n* = 63), 6.03 ± 0.32 (*n* = 41), 7.07 ± 0.26 (*n* = 24) years old at the health care check-ups at ages 1, 2, 3, 4, 5, 6 and 7 years, respectively. Therefore, an estimation of the weight and height at a given anniversary was calculated based on the exact check-up date (((weight_check-up_ − birth weight_kg_)/(check-up – birthday-date)) × (365 × age_check-up_))/1000. A similar procedure was used for calculating height at the exact date of the anniversary. These date-corrected estimates of weight and height were used in all descriptives, as well as when age-standardized BMI_z_ scores were calculated for further statistical analyses. For each child, an age adjusted BMI_z_ score was calculated at the ages of 2, 3, 4, 5 years and at the age of the tests of the present study (5–7 years) using a Children’s BMI Group Calculator—Metric Version, provided by the Centers for Disease Control and Prevention [33]. Overweight was defined, according to the Centers for Disease Control and Prevention, as BMI ≥ 85 percentile in the reference group. There are no international age-adjusted BMI references for children under two years old, therefore, we did not calculate nor use BMI values for ages under two.

### 2.5. Parental Education

We asked parents to evaluate the highest achieved educational level on the scale from zero to four (zero = elementary school, one = secondary school, two = high school, three = vocational or intermediate degree, four = polytechnic or university degree). A mean of the highest educational level of parent(s) was calculated and used for analyses. Finally, a dichotomous variable of “higher education” (value four) and “no higher education” (values from zero to three) was formed and used in descriptives and analyses.

### 2.6. Statistical Analyses

All analyses were conducted for all children and separately for both sexes with the Statistical Package for the Social Sciences statistics software (IBM SPSS Statistics versions 24, SPSS Finland, Espoo, Finland). Means, standard deviations, and ranges of descriptive statistics are presented and differences between sexes were tested by independent samples *T*-tests for continuous variables (age, weight, height, BMI_z_ score, MC, physical activity) and by chi-square tests for binomial variables (overweight, education). The prevalence of overweight among the children and higher degree educational status in the children’s parents are also reported. Prior to conducting further analyses, the relevant assumptions of statistical analyses were tested.

Correlations between MC, age, MVPA, education level of parents, birth weight, and BMI_z_ score at ages 2, 3, 4, 5 years and BMI_z_ at middle childhood in all children were studied by Pearson’s correlation coefficients. Forced-entry linear regressions were utilized for evaluating the variation explained in the MC by the independent predictors. The predictors of MC were chosen for the model based on earlier research evidence (age, sex, MVPA, BMI) and based on the theoretically meaningful roles on the MC (education level of parents, child’s birth weight). A linear hierarchical regression was utilized to examine the independent predictive effect of early years’ BMI_z_ scores on MC. Accordingly, hierarchical regression models were built with covariates (age, sex, MVPA, education, and birth weight) at the first level, and the BMI_z_ scores at ages 2, 3, 4, 5 years and at middle childhood, each in a separate model, at the second level. In all analyses, the level of significance was set to *p* < 0.05.

## 3. Results

Table 1 describes the characteristics of the study sample. Girls and boys were equally represented and their age ranged between 5 and 7 years. The sample consisted of mostly normal-weight children. Parents of the children consisted mostly of those having a higher-level educational degree (university or polytechnic). Boys outperformed girls in the hopping for height MC subtest (*t* = 3.29, *p* < 0.01), although there were no sex differences in the total MC. Boys accumulated more light (*t* = 3.86, *p* < 0.001), moderate (*t* = 3.68, *p* < 0.001), vigorous (*t* = 2.60, *p* < 0.05), and MVPA (*t* = 3.29, *p* < 0.01) minutes per day compared to girls.

**Figure 1:**
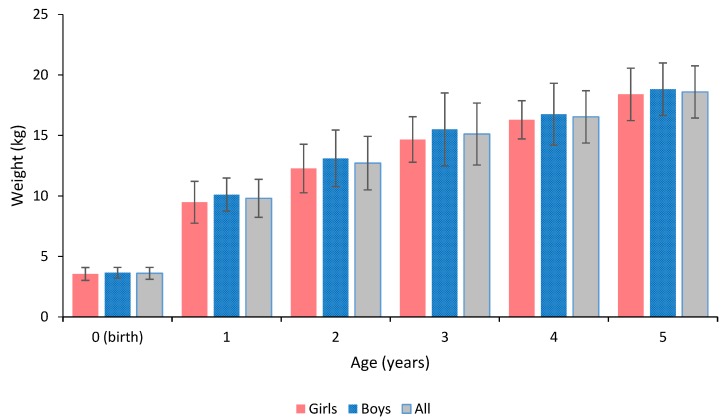
Descriptive statistics of weight history of the children.

**Figure 2:**
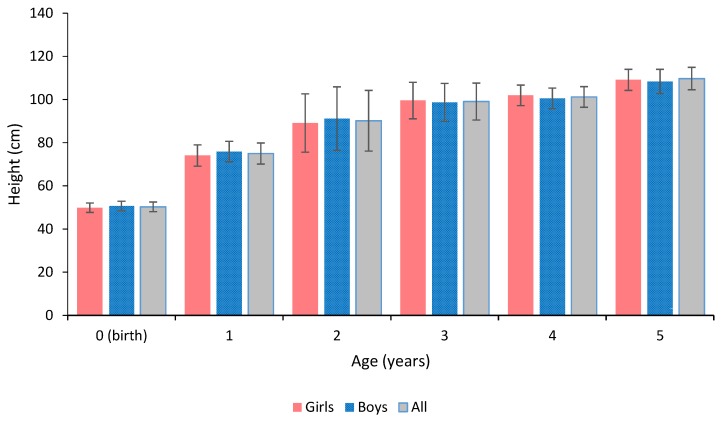
Descriptive statistics of height history of the children.

The standard deviation of weight approximately tripled from the birth weight to the age of 1 year and quadrupled to the latter age points (Figure 1). The standard deviation of height was generally highest at the age of 2 years (Figure 2). There were no significant sex differences in the birth weight or height, nor in the weight or height at ages of 1, 2, 3, 4, or 5 years.

Age and MVPA at middle childhood positively correlated with MC in general (Table 2). When correlations were tested separately for boys and girls, age correlated positively and BMI_z_ score negatively with MC in girls at middle childhood. From the age of 2 years onwards, BMI_z_ score positively correlated with the following year’s BMI_z_ score in girls. The same was found in boys from the age of 3 years onwards. The correlations between BMI_z_ scores at different ages were higher among the girls compared to the boys. In addition, birth weight positively correlated with the BMI_z_ score at the age of 4 years and at middle childhood in general.

The regression analysis revealed that age, sex, parents’ education level, birth weight, MVPA, and BMI_z_ score at middle childhood together explained significantly and 17.7% of the variation in MC (Table 3). However, only age, MVPA, and BMI_z_ score at middle childhood were statistically significant predictors of MC.

The predictive value of BMI_z_ score at the different ages on the MC was then tested by taking the effect of age, parental education, birth weight, and MVPA into account (Table 4). BMI_z_ score at middle childhood explained approximately 9% of the variation in MC in general (*p* = 0.014) and approximately 12% of the variation in MC in girls (*p* = 0.042). In addition, BMI_z_ score at ages of 4 and 5 years explained approximately 6% (n.s.) and 7% (n.s.) of the variation in MC in girls, respectively. BMI_z_ scores did not show any predictive value in MC in boys.

## 4. Discussion

The main aim of this study was to examine the association between BMI in early childhood and MC in middle childhood. A statistically significant association was not found between BMI in the early years and MC later in childhood, though a nascent and insignificant trend of association was found in girls, from the age of 4 years onwards. In line with recent findings in the field [8], BMI and MC were cross-sectionally associated in middle childhood in girls only. Previous research has indicated that the relationship between BMI and MC strengthens from the age of 6 until 11 years [10]. This study is, to our knowledge, the first inspecting this relationship under the age of 6 years, although retrospectively. The results suggest that BMI in early childhood does not predict MC in middle childhood in boys, but a weak predictive association may exist in girls, although it was found to be insignificant in the present study.

In contrast to the girls, neither the concurrent BMI nor BMI in the early years showed a trend of association with MC in boys. Regarding PA, the time spent at MVPA showed a trend toward an association with MC in boys but not in girls. These findings are in line with the literature, which has shown that in addition to the fitness, muscular strength, and endurance [1,2], the time spent at MVPA is among the most consistent health-related correlates of MC in children [3]. Overall, the results suggest that essentially different mechanisms influencing motor development may exist in girls and boys in early childhood. This suggests a need for sex-specific early interventions for enhancing motor development, PA, and preventing excess weight gain. Of relevance, excess weight gain increases remarkably the risk of overweight and obesity later in life [11], and children with low MC may be at higher risk of overweight, obesity, and related health risks later in adolescence [6,8].

High BMI and body fatness have been shown to associate with low movement proficiency in 6 to 11-year-old girls but not in boys [7]. The present study is in line with these findings by showing a significant inverse association between BMI and MC at the age 5 to 7 years in girls. The present study suggests that the BMI development from the age of 4 years onwards may already predict, although it was found to be statistically insignificant, the development of MC in girls. Presumably, girls with greater body weight gain during early childhood are not participating as much in physical activities developing MC as the girls with smaller body weight gain. It is possible that carrying a relatively large amount of body weight makes it physiologically more difficult to participate in physical activities typical to age and developmental level. However, there is no evidence until now showing that BMI and MC, nor that BMI and PA, would go hand by hand during early childhood. While the present study supports earlier findings of the association between MC and concurrent BMI [2], the results suggest that this relationship may start to emerge already during the first years of life in girls.

Further studies should investigate if trajectories of BMI and motor development are parallel in early childhood or if there exists a causal-relationship between these factors. The limited body of evidence in the field suggests that if one is not improving motor competence or fitness between the ages of 5 and 10 years, there exists an added risk for overweight and obesity [8,34]. On the other hand, the causal relationship between BMI and MC has been shown to be bidirectional at the ages of 5 to 13 years, so that children’s weight status negatively influences the development of MC and vice versa [6]. Very low or high body fat percentage accompanied with poor MC is known to associate with poor cognition in 6 to 8-year-old children [35]. Therefore, it may be that overweight and obesity are related to neurological difficulties in motor learning. Excess weight gain may hinder motor development via deteriorated neurocognitive functionality [36]. On the other hand, several behavioral (e.g., nutrition, physical activity, and types of sedentary behavior) and environmental (e.g., parental BMI) factors interact with child’s BMI development throughout childhood [17]. Therefore, behavioral and environmental factors may mediate the association between BMI and MC. In essence, it is likely that behavioral and environmental factors augment either negative or positive trajectories of BMI status, PA, motor development, and fitness in childhood, and therefore, all these factors should be taken into account as a whole in future studies.

Lastly, the use of more accurate measures of body composition would strengthen the likelihood of detecting significant relationships with MC in children. For instance, waist-for-height ratio has been successfully used as a measure of body composition in studies considering motor development [37] and cardiovascular disease risk factors [38] in children. Ideally, the examination of fat-free mass, such as muscle and bone, or body fat percentage would guarantee even more accurate measures of body fatness [39], and would therefore strengthen the power to detect associations between body composition and MC.

## 5. Study Strengths and Limitations

The strengths of this study relate to the use of a validated assessment protocol for MC and objective PA measurements. Additionally, the retrospective information on body weight and height in the early years was based on measurements conducted by qualified public health nurses in child health care centers. Limitations of the study relate to the relatively small sample size, the lack of obese children, and the relatively high educational level of the parents. The small sample size may decrease the power to detect statistically significant results in general. The statistical power was further reduced when sexes were analyzed separately. Variations in body weight and height were found to widen along the age in the present study, and this is in line with nationally representative samples [40]. In addition, the prevalence of overweight children was within the national ranges [41], but the lack of obese children in the study sample possibly weakened the statistical power to detect an association between BMI in the early years and MC at middle childhood. In addition, BMI has limitations for assessing body composition as it does not take into account individual build, such as the amount of muscles and fat in the body. Therefore, the use of waist-to-height ratio, body fat percentage, or other more accurate measures of body composition would strengthen the power to detect significant associations with MC. Additionally, the study sample consisted mostly of highly educated parents, which may conceal some effects that excess body weight accumulation may have. In essence, highly educated parents are more likely to support their children in PA than less educated parents [42], and therefore physical activity parenting may counteract the detrimental influences that excess weight gain may have on the development of MC. Overall, the results of the present study should be replicated with a more representative sample to confirm the validity of the presented findings.

## Figures and Tables

**Table 1:** Descriptive statistics of the participants.

Variables	Girls	Boys	All
	Mean ± SD (Range)	Mean ± SD (Range)	Mean ± SD (Range)
N	32	32	64
Age	6.52 ± 1.04 (2.77)	6.15 ± 1.02 (2.92)	6.33 ± 1.04 (2.92)
Height (cm)	122.68 ± 7.98 (27.4)	121.15 ± 6.68 (26.9)	121.94 ± 7.36 (29)
Weight (kg)	23.62 ± 3.98 (12.8)	23.23 ± 3.53 (14.4)	23.43 ± 3.74 (14.4)
Body mass index z-score	15.65 ± 1.66 (8.31)	15.73 ± 0.97 (3.84)	15.69 ± 1.36 (8.31)
Overweight (%)	4 (12.5)	1 (3.3)	5 (8.1)
Higher education, parents (%)	27 (84.4)	25 (78.1)	52 (81.3)
Motor coordination ^#^	108.56 ± 13.50 (61)	113.31 ± 15.24 (65)	110,94 ± 14,48 (66)
Walking backwards	99.56 ± 12.65 (57)	96.66 ± 11.86 (47)	97.61 ± 12.20 (57)
Hopping for height	100.38 ± 12.31 (51)	112.50 ± 16.83 (63) **	106.44 ± 15.85 (71)
Jumping sideways	116.06 ± 15.16 (71)	121.25 ± 15.49 (59)	118.66 ± 15.43 (73)
Moving sideways	112.13 ± 14.81 (59)	111.13 ± 14.02 (63)	111.63 ± 14.31 (68)
Physical activity (mins/day)			
Sedentary	632.35 ± 55.99 (248.86)	622.6 ± 52.67 (218.85)	627.47 ± 54.13 (272)
Light	32.97 ± 7.84 (32.85)	43.4 ± 12.81 (58.4) ***	38.18 ± 11.77 (59.2)
Moderate	21.05 ± 6.18 (27.8)	29.16 ± 10.58 (49.69) ***	25.1 ± 9.51 (49.69)
Vigorous	18.47 ± 10.18 (46.47)	27.1 ± 15.44 (69.86) *	22.78 ± 13.68 (69.86)
Moderate to vigorous physical activity	39.52 ± 15.08 (63.96)	56.25 ± 23.96 (104.75) **	47.89 ± 21.57 (104.75)

Data are means ± SD or *n* (%). * Significant sex difference at the level of *p* < 0.05; ** Significant sex difference at the level of *p* < 0.01; *** Significant sex difference at the level of *p* < 0.001; ^#^ General motor quotient of the Körperkoordination Test für Kinder.

**Table 2:** Correlation matrix for motor coordination, age, moderate-to-vigorous physical activity, parental education, birth weight, BMI_z_ history, and prevailing BMI_z_ score.

Girls (*n* = 18–32)	(1)	(2)	(3)	(4)	(5)	(6)	(7)	(8)	(9)	(10)
(1) MC	1.00									
(2) Age	0.446 *	1.00								
(3) MVPA	0.137	0.047	1.00							
(4) Education	0.076	−0.099	−0.158	1.00						
(5) Birth weight	−0.186	−0.045	−0.256	−0.036	1.00					
(6) BMI_z_ at age 2	−0.093	−0.253	−0.132	0.036	0.119	1.00				
(7) BMI_z_ at age 3	−0.156	−0.103	−0.055	−0.190	0.215	0.809 ***	1.00			
(8) BMI_z_ at age 4	−0.314	−0.118	0.047	−0.024	0.322	0.440 *	0.631 **	1.00		
(9) BMI_z_ at age 5	−0.304	−0.107	0.141	−0.085	0.077	0.487 *	0.677 **	0.845 ***	1.00	
(10) BMI_z_ at age 5–7	−0.368 *	−0.043	0.124	−0.055	0.310	0.285	0.220	0.674 ***	0.763 ***	1.00
**Boys (*n* = 23–32)**	**(1)**	**(2)**	**(3)**	**(4)**	**(5)**	**(6)**	**(7)**	**(8)**	**(9)**	**(10)**
(1) MC	1.00									
(2) Age	0.220	1.00								
(3) MVPA	0.313	0.146	1.00							
(4) Education	−0.145	−0.056	−0.158	1.00						
(5) Birth weight	0.126	0.061	−0.342	−0.246	1.00					
(6) BMI_z_ at age 2	−0.238	−0.191	−0.451 *	0.223	−0.138	1.00				
(7) BMI_z_ at age 3	−0.053	−0.035	−0.006	0.212	−0.181	0.287	1.00			
(8) BMI_z_ at age 4	−0.020	0.002	0.026	−0.258	0.279	−0.358	0.322	1.00		
(9) BMI_z_ at age 5	0.211	−0.188	0.172	−0.189	0.262	−0.213	0.490 *	0.697 ***	1.00	
(10) BMI_z_ at age 5–7	−0.043	0.318	0.075	−0.305	0.159	−0.079	0.258	0.426 **	0.516 **	1.00
**All (*n* = 41–64)**	**(1)**	**(2)**	**(3)**	**(4)**	**(5)**	**(6)**	**(7)**	**(8)**	**(9)**	**(10)**
(1) MC	1.00									
(2) Age	0.286 *	1.00								
(3) MVPA	0.291 *	0.021	1.00							
(4) Education	−0.061	−0.060	−0.175	1.00						
(5) Birth weight	−0.016	−0.017	−0.229	−0.144	1.00					
(6) BMI_z_ at age 2	−0.161	−0.226	−0.257	0.134	−0.003	1.00				
(7) BMI_z_ at age 3	−0.071	−0.069	0.024	0.043	0.000	0.509 **	1.00			
(8) BMI_z_ at age 4	−0.142	−0.090	0.100	−0.143	0.316 *	0.070	0.458 **	1.00		
(9) BMI_z_ at age 5	0.002	−0.170	0.210	−0.155	0.189	0.145	0.560 ***	0.782 ***	1.00	
(10) BMI_z_ at age 5–7	−0.221	−0.075	0.097	−0.146	0.260 *	0.148	0.231	0.593 ***	0.652 ***	1.00

MC = Motor coordination; MVPA = Moderate-to-vigorous physical activity; Education = parental education; BMI_z_ = age-adjusted body mass index. * Significant at the level of *p* < 0.05; ** Significant at the level of *p* < 0.01; *** Significant at the level of *p* < 0.001.

**Table 3:** Linear regression models predicting motor coordination in girls, boys, and in all children.

Variables	Girls		Boys		All	
	Standardized β	*p*	Standardized β	*p*	Standardized β	*p*
Age	0.437	**0.011**	0.158	0.426	0.309	**0.013**
Sex					0.120	0.371
Education	0.124	0.763	−0.023	0.909	0.002	0.990
Birth weight	0.001	0.997	0.319	0.140	0.141	0.283
MVPA	0.182	0.299	0.397	0.062	0.299	**0.036**
BMI_z_ at age 5–7 years	−0.369	**0.042**	−0.181	0.377	−0.315	**0.014**
R^2^	0.365		0.215		0.260	
Adjusted R^2^	0.238		0.051		0.177	
*P*	**0.035**		0.291		**0.010**	

Bold font denotes significance at *p* < 0.05.

**Table 4:** Standardized regression coefficients of motor coordination with body mass index z-scores at ages 2, 3, 4, 5 years and at middle childhood independent of age, parental education, birth weight, and the level of moderate-to-vigorous physical activity.

Outcome	BMI_z_ at Age 2		BMI_z_ at Age 3		BMI_z_ at Age 4		BMI_z_ at Age 5		BMI_z_ at Age 5–7	
	Standardized β (95% CI)	*p*	Standardized β (95% CI)	*p*	Standardized β (95% CI)	*p*	Standardized β (95% CI)	*p*	Standardized β (95% CI)	*p*
MC-girls ^a^	0.051 (−0.360 to 0.463)	0.797	−0.025 (−0.463 to 0.414)	0.906	−0.272 (−0.688 to 0.143)	0.187	−0.266 (−0.657 to 0.125)	0.172	−0.369 (−0.014 to −0.723)	**0.042**
MC-boys ^a^	−0.102 (−0.567 to 0.362)	0.652	0.072 (−0.300 to 0.444)	0.690	−0.063 (−0.477 to 0.351)	0.757	0.111 (−0.308 to 0.529)	0.588	−0.181 (−0.596 to 0.234)	0.377
MC-all ^b^	−.059 (−.344 to 0.226)	0.679	−0.057 (−0.319 to 0.204)	0.659	−0.188 (−0.466 to 0.090)	0.181	−0.042 (−0.321 to 0.237)	0.763	−0.315 (−0.563 to −0.066)	**0.014**

MC = Motor coordination. Boldface font denotes significance at *p* < 0.05. ^a^ Adjusted for age, parental education, birth weight, and moderate-to-vigorous physical activity; ^b^ Adjusted for sex, age, parental education, birth weight, and moderate-to-vigorous physical activity.
